# scGenoByte: a GenoByte embedding transformer with biological priors for cell type annotation

**DOI:** 10.1093/bib/bbag369

**Published:** 2026-07-06

**Authors:** Jiongsen Yao, Yong Xu, Jinjin Ma, Wenjun Shen, Si Wu

**Affiliations:** School of Computer Science and Engineering, South China University of Technology, Building B3, 382 Waihuan East Road, Guangzhou Higher Education Mega Centre, Panyu District, Guangzhou 510006, Guangdong, China; School of Computer Science and Engineering, South China University of Technology, Building B3, 382 Waihuan East Road, Guangzhou Higher Education Mega Centre, Panyu District, Guangzhou 510006, Guangdong, China; The Institute of Future Health, South China University of Technology, Guangzhou International Campus, 777 Xingye Avenue East, Panyu District, Guangzhou 511442, Guangdong, China; Department of Bioinformatics, Shantou University Medical College, 22 Xinling Road, Jinping District, Shantou 515041, Guangdong, China; School of Computer Science and Engineering, South China University of Technology, Building B3, 382 Waihuan East Road, Guangzhou Higher Education Mega Centre, Panyu District, Guangzhou 510006, Guangdong, China

**Keywords:** single-cell RNA-seq, cell type annotation, biological priors, masked autoencoder, foundation model

## Abstract

Effective cell representation learning is crucial for accurate cell annotation and the deciphering of cellular heterogeneity in single-cell RNA sequencing (scRNA-seq) analysis. Current foundation models have achieved superior performance compared with traditional methods. However, due to data sparsity and the complexity of model, existing methods often compromise by selecting highly variable genes or filtering for nonzero expressions, which discard potentially significant genes. Thus, modeling the complete transcriptome for cell representation remains computationally challenging; we present scGenoByte, a unified framework designed to enhance cell representation learning through biologically informed full-gene modeling. To enable efficient modeling of the full transcriptome, we design GenoBytes, biologically coherent units that are constructed by leveraging biological priors in terms of protein–protein interaction network and gene paralogy network. Furthermore, considering that the information of protein and pathway is critical for analyzing cell functions and representation, scGenoByte encapsulates biological priors by harmonizing GenoByte embeddings with protein representations and leveraging an auxiliary task of pathway activity prediction to impose pathway-guided regularization. Extensive results on eight datasets have shown that scGenoByte achieves better performance than competing methods, which confirms the efficacy of combining full-gene context with biological priors.

## Introduction

The advent of single-cell RNA sequencing (scRNA-seq) has revolutionized our understanding of cellular heterogeneity by enabling transcriptomic profiling at individual cell resolution [1, 2]. This technology not only facilitates the characterization of cells, but explains complex functional states and pathological processes. However, deriving robust cellular representations from scRNA-seq data remains inherently challenging due to technical noise and data sparsity [3–5]. To gain better cellular representations from scRNA-seq data, early works have established the utility of latent spaces for cell characterization leveraging methods such as statistical clustering [6], reference-based projection [7, 8], and generative probabilistic modeling [9, 10].

As datasets scale to millions of cells, deep learning paradigms have emerged for capturing gene–gene interactions, including graph-based methods [11–13] and transformer-based methods [14, 15]. Particularly, to learn universal cell embeddings, single-cell foundational models such as scBERT [16], scGPT [17], and scFoundation [18] learn the interaction of genes from massive unlabeled data, achieving excellent performance in the cell annotation task. However, applying transformers to full transcriptome faces a critical scalability bottleneck, as the self-attention mechanism treats every gene as a distinct token, which makes holistic genomic modeling computationally intractable.

To reconcile high-dimensional transcriptomics with the complexity of architecture, existing methods resort to reductionist strategies that compromise representation quality. The first strategy relies on aggressive gene selection, including selecting only nonzero expression genes or highly variable genes. While this strategy efficiently reduces dimensions, some genes that serve as critical determinants of cell identity are ignored. The second strategy aggregates genes into non-biological patches or sliding windows [19, 20]. Such structures risk severing intrinsic functional connections [21, 22], forcing models to learn from biologically incoherent units and limiting the ability to capture high-order biological semantics.

Furthermore, while learning representation solely on transcriptomic data allows capturing statistical correlations, it misses functional information of cellular states. Thereby, recent works have attempted to incorporate biological priors for cell representation. For example, GenePT encodes gene functional semantics through large language models [23], UCE captures evolutionary conservation via protein-centric embeddings [24], and scGraph and CellPLM incorporate topological molecular networks or multicellular interactions for representation [25, 26]. Considering biological functions are ultimately executed by proteins operating within coordinated signaling pathways [27], we hypothesize that a robust cellular representation should not only capture the full spectrum of gene expression but also actively align with the functional landscape defined by the biological priors such as protein–protein network and pathways-level information.

**Figure 1:**
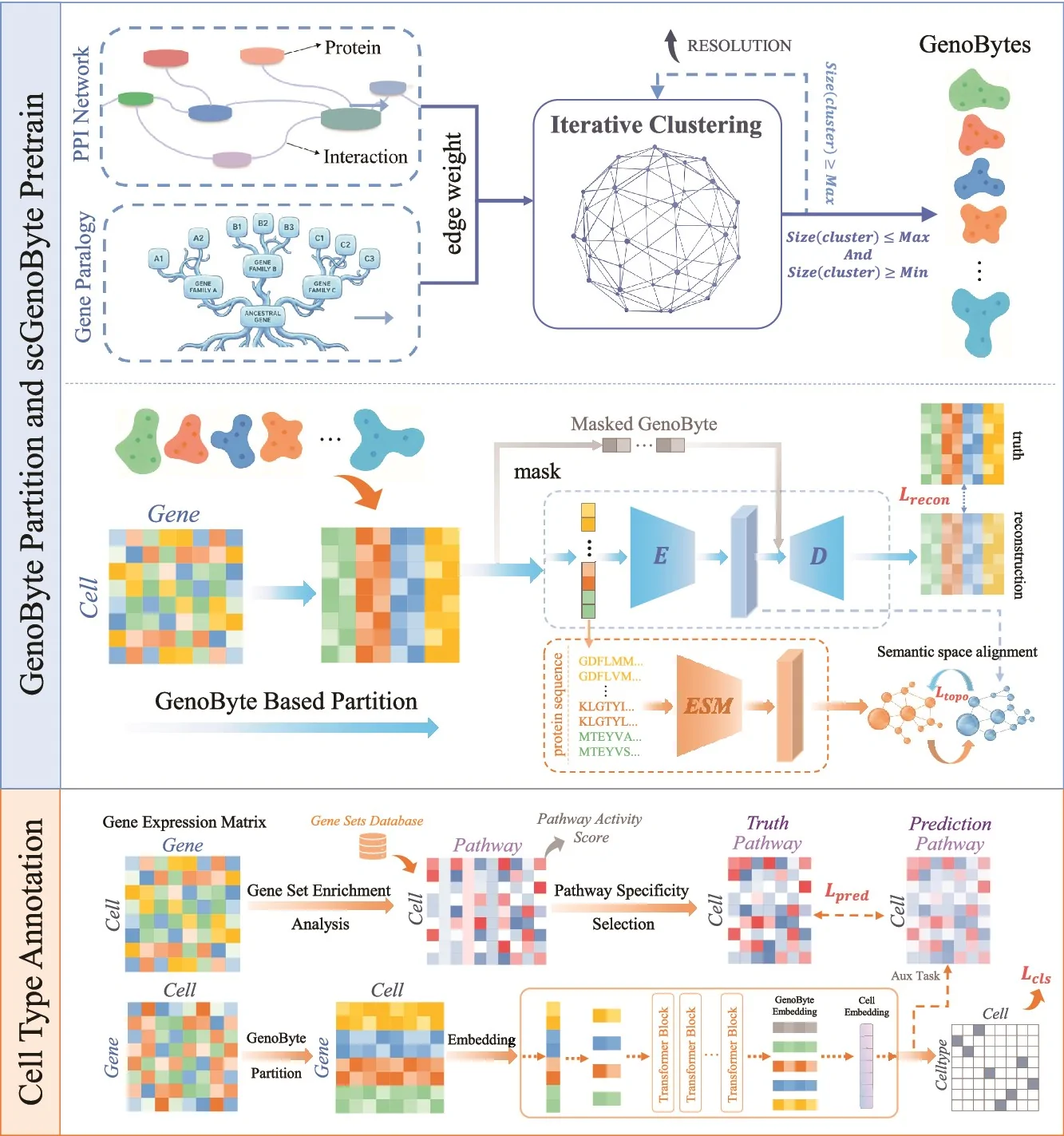
Schematic overview of the scGenoByte framework.Top: GenoByte construction via resolution-adaptive iterative clustering on the composite graph of PPI and gene paralogy networks. Middle: proteomic-consistent masked pretraining, incorporating masked GenoByte modeling ($\mathcal{L}_{recon}$) and topological semantic alignment ($\mathcal{L}_{topo}$) to anchor transcriptomic embeddings to protein evolutionary priors. Bottom: pathway-guided fine-tuning, where cell type annotation is jointly optimized with an auxiliary pathway activity prediction task to enforce functional regularization.

In this work, we propose scGenoByte, a unified framework designed to enhance cell representation learning through biologically informed full-gene modeling, as shown in Fig. 1. In scGenoByte, tokens are not restricted to biologically arbitrary patches or limited to nonzero expression genes. They are structured as biologically coherent units termed GenoBytes, which are derived from resolution-adjusted iterative clustering over protein–protein interaction (PPI) and gene paralogy networks. To acquire universal cell characterization capabilities, scGenoByte adopts a masked genes reconstruction framework and tailors the GenoByte representation learning paradigm to address the issues of data zero-inflation and computational bottlenecks. Furthermore, scGenoByte embeds GenoByte in alignment with the semantics of protein evolution and utilizes pathway-guided regularization as an auxiliary task to enhance representation from rich biological prior knowledge. This ensures GenoByte embeddings learned by scGenoByte not only maintain a topological structure in the representation space, which is consistent with the protein sequence representation space, but also functionally align with the underlying biological mechanisms.

## Method

### Overall framework

The scGenoByte framework presents a novel framework for biologically informed full-gene modeling. As shown in Fig. 1, the process begins with the construction of GenoBytes, during which we reorganize the high-dimensional gene expression space GenoBytes with biological consistency. Subsequently, during the pretraining phase, we leverage a masked autoencoder framework to simultaneously reconstruct the masked GenoByte expression and harmonize protein evolutionary semantics [28]. Ultimately, the annotation of cell types and the auxiliary task for pathway activity are synergistically optimized.

### GenoByte construction via iterative clustering

To reconcile the high dimensionality of the full transcriptomic landscape with the computational constraints of transformer architectures, we propose a biologically informed partitioning strategy. This approach organizes genes into functionally cohesive GenoBytes, ensuring efficient full transcriptome modeling while preserving biological fidelity. The construction process relies on building a composite interaction graph and partitioning it using a resolution-adjusted iterative clustering algorithm.

**Biological interaction graph construction.** We define the gene interaction graph as $\mathcal{G} = (\mathcal{V}, \mathcal{E}, \mathcal{W})$, where $\mathcal{V} = \{g_{1}, \dots , g_{M}\}$ represents the set of *M* genes. The edge set $\mathcal{E}$ and the corresponding weight matrix $\mathcal{W}$ are derived by integrating PPI and gene paralogy networks. Specifically, the weight $w_{ij}$ between gene $g_{i}$ and $g_{j}$ is defined as a weighted sum of the information from PPI and the gene paralogy network: 


(1) \begin{align*}& w_{ij} = S_{PPI}(i, j) + \alpha \cdot S_{Paralogy}(i, j),\end{align*}

where $S_{PPI}$ denotes the PPI score obtained from scNET [29], and $S_{Paralogy}$ represents the homology identity score derived from the Ensembl database [30] via the BioMart interface [31, 32] to capture homology-based functional associations. *α * is a hyperparameter controlling the contribution of homologous relationships. Conceptually, this construction establishes the topology of the biological environment, where the weighted edges $w_{ij}$ serve as functional affinities that dictate the model’s navigation through the high-dimensional transcriptomic landscape. Rather than increasing model complexity, the construction of biological interaction graph provides a structured abstraction of the full transcriptome, allowing over 20 000 genes to be reorganized into biologically coherent GenoBytes. From a representation-learning perspective, Equation (1) defines the topology of a biological state space, while the subsequent iterative clustering acts as a state-abstraction mechanism that maps noisy gene-level observations into compact functional units. The abstraction improves robustness to sparsity and technical noise by encouraging partitions that preserve biological fidelity and functional coherence.

**Resolution-adjusted iterative clustering.** To organize the genes $\mathcal{V}$ into biologically coherent GenoBytes based on the topology of graph $\mathcal{G}$, we devise a resolution-adaptive iterative clustering strategy rooted in the leiden algorithm [33]. Conventional community detection algorithms typically yield clusters with substantial size heterogeneity. To mitigate this, our algorithm dynamically adjusts the resolution to ensure the size of each GenoByte $\mathcal{B}_{k}$ remains within a theoretically optimal range $|\mathcal{B}_{k}| \in [Size_{min}, Size_{max}]$. The process initiates with a baseline resolution *γ = 1.0*. For any resultant cluster exceeding the upper bound $Size_{max}$, the algorithm recursively decomposes the subgraph using an adaptive resolution increment ($\Delta \gamma = 0.5$) until all partitions satisfy the valid size criteria, which guarantees GenoBytes encapsulate rich local information. Furthermore, to ensure robustness, we execute the algorithm across multiple random seeds and select the partition that maximizes the modularity score, ensuring an optimized structure not contingent on any single initialization. The selection of $Size_{max}$ in the regime of 16 represents a strategic trade-off between biological representation quality and computational tractability. As shown in Supplementary Materials S4.2, increasing the GenoByte size from 8 to 16 yields only a negligible change in accuracy while sharply reducing computational cost, whereas larger GenoByte sizes substantially degrade performance. A reasonable choice of $Size_{max}$ allows scGenoByte to achieve optimal performance while effectively overcoming the quadratic complexity typically associated with full-transcriptome modeling. More details about the algorithm flow are in Supplementary Materials S2.

### Protein-consistent masked pretraining

Upon the foundation of GenoBytes, scGenoByte implements a synergistic self-supervised pretraining paradigm. This phase aims to cultivate robust cellular representations by not only recovering masked transcriptomic features but also simultaneously imposing cross-modality consistency constraints.

**Masked GenoByte modeling.** To extract robust semantic features from the inherent sparsity and noise in scRNA-seq data, we adopted the masked autoencoder paradigm. The input gene expression profile is formalized as a sequence of GenoBytes $\mathcal{B} = \{\mathcal{B}_{1}, \mathcal{B}_{2}, \dots , \mathcal{B}_{N}\}$. Then we project the GenoBytes into a latent feature space augmented with positional encoding, which ensures that scGenoByte can distinguish each unit. For each GenoByte $\mathcal{B}_{i}$, the input embedding $z_{i}^{(0)}$ is computed as 


(2) \begin{align*}& z_{i}^{(0)} = W_{in} \mathcal{B}_{i} + p_{i},\end{align*}

where $W_{in}$ denotes the learnable linear projection matrix and $p_{i}$ represents the learnable positional embedding for the *i*th unit, which is used to distinguish the same pattern of expression in different gene functional modules.

We apply a stochastic masking strategy by sampling a subset of indices $\mathcal{M}$ to be masked. The sequence of visible embeddings is then processed by the transformer encoder *E(· )* to capture global context, yielding the latent GenoByte representations $H_{\mathcal{B}}$. Subsequently, a lightweight decoder *D(· )* maps these latent features back to the original expression space. These processes are formalized as 


(3) \begin{align*} H_{\mathcal{B}} &= E(\tilde{Z}^{(0)}), \end{align*}

(4) \begin{align*} \hat{\mathcal{B}}_{i} &= D(h_{i}), \end{align*}

where $\tilde{Z}^{(0)}$ represents the unmasked input sequence, and $h_{i} \in H_{\mathcal{B}}$ corresponds to the latent vector at position *i*. The reconstruction objective is minimized via the mean squared error: 


(5) \begin{align*}& \mathcal{L}_{recon} = \mathbb{E}_{x \sim \mathcal{D}, \mathcal{M} \sim p(\mathcal{M})} \left[ \frac{1}{|\mathcal{M}|} \sum_{i \in \mathcal{M}} \| \mathcal{B}_{i} - \hat{\mathcal{B}}_{i} \|^{2}_{2} \right],\end{align*}

where $\hat{\mathcal{B}}_{i}$ denotes the reconstructed expression vector. This objective incentivizes the model to infer missing transcriptional signals by learning the high-order gene–gene correlations and functional dependencies intrinsically encoded within the GenoBytes.

**Protein-informed semantic alignment.** While masked GenoByte modeling efficiently captures transcriptomic statistical dependencies, it operates without explicit guidance from the knowledge of proteins. To bridge this modality gap, we introduce a semantic alignment mechanism that anchors transcriptomic representations to proteomic evolutionary priors. We obtain reference protein sequences via the Ensembl database and use ESM-2 [34] for representation. For each gene, we calculate the center of its reference representation to serve as its unique protein-level representation. Then during pretraining, we construct a proteomic anchor $H_{\mathcal{P}}$ for each GenoByte by embedding aggregation.

In addition, to avoid the fragility brought by the rigid one-to-one mapping, we introduce topological isomorphism between the two modalities, ensuring the pairwise geometric relationships of gene representations learned are consistent with those in the protein space. We generate a random permutation *π * of the batch indices and compute the structural distance vectors: 


(6) \begin{align*}& \begin{split} d_{\mathcal{B}}[i] &= 1 - \cos(h^{\mathcal{B}}_{i}, h^{\mathcal{B}}_{\pi(i)}), \\ d_{\mathcal{P}}[i] &= 1 - \cos(h^{\mathcal{P}}_{i}, h^{\mathcal{P}}_{\pi(i)}). \end{split}\end{align*}

Then the topological consistency loss is defined as the mean squared error between these distance vectors: 


(7) \begin{align*}& \mathcal{L}_{topo} = \mathbb{E}_{B, \pi} \left[ \frac{1}{N} \big\| d_{\mathcal{B}} - d_{\mathcal{P}} \big\|^{2}_{2} \right],\end{align*}

where $h_{i}$ and $h_{\pi (i)}$ denote the embedding of the *i*th GenoByte and its randomly paired counterpart. This objective constrains the potential space of GenoBytes, aligning it with the evolutionary information of protein sequences, and promotes the model’s understanding of different GenoBytes.

**General representation learning objective.** Consequently, the unified pretraining parameters *Θ * are optimized by solving the following minimization problem: 


(8) \begin{align*}& \min_{\theta_{pre}} \mathcal{L}_{\mathrm{recon}} + \lambda_{\mathrm{topo}} \mathcal{L}_{\mathrm{topo}},\end{align*}

where $\theta _{pre}$ denotes the set of learnable parameters in the scGenoByte framework, and $\lambda _{topo}$ is a hyperparameter that modulates the strength of the proteomic structural constraint.

### Cell type annotation with the auxiliary pathway activity prediction task

Rather than treating annotation as a simple mapping task, we seek to ensure that the learned cellular embeddings remain faithful to biological reality. To this end, we introduce a pathway-regularized strategy for cell type annotation.

**Discriminative cell identity learning.** The fundamental goal of this phase is to translate the learned transcriptomic representations into discrete cell identity labels. We attach a linear classification head to the aggregated cell-level embedding $h_{cell}$ generated by the transformer encoder. We minimize the cross-entropy loss to sharpen the decision boundaries between distinct cell populations: 


(9) \begin{align*}& \mathcal{L}_{cls} = \mathbb{E}_{(x, y) \sim \mathcal{D}} \left[ - \sum_{c=1}^{C} y_{c} \log(\hat{y}_{c}(x)) \right],\end{align*}

where $y_{c}$ and $\hat{y}_{c}$ denote the one-hot ground-truth label and the predicted probability for class *c*, respectively.

**Mechanism-aware auxiliary regularization.** Standard classification often risks overfitting to batch effects or technical noise. To mitigate this and augment the learned representations with biological semantics, we introduce pathway functional state inference as an auxiliary supervision task. Let $t \in \mathbb{R}^{P}$ be the ground-truth pathway activity vector obtained through single-sample Gene Set Enrichment Analysis (ssGSEA) using Reactome [35] on the training data. Specifically, we utilized the scoring algorithm implemented in Python package gseapy [36] to compute the per-cell activity scores for Reactome pathways. Here, the activity score is defined as the enrichment score derived from the rank-transformed gene expression distribution within each cell, serving as a proxy for functional activation. To ensure the discriminative power of the auxiliary task, we further filtered these pathways using the Wilcoxon rank-sum test, retaining only those significantly enriched in specific cell types. The model is tasked with regressing these activity scores based on cell embedding $h_{cell}$. This enhances the ability of scGenoByte to effectively translate transcriptomic patterns into high-level functional states.

Considering the potential outliers in pathway activity scores, we introduced the Smooth $\ell _{1}$ Loss. This provides a robust regression target with lower sensitivity to abnormal activity peaks: 


(10) \begin{align*}& \mathcal{L}_{path} = \mathbb{E}_{(x, t) \sim \mathcal{D}} \left[ \frac{1}{P} \sum_{j=1}^{P} \phi(t_{j} - \hat{t}_{j}(x)) \right],\end{align*}

where $\phi (\cdot )$ denotes smoothing kernel [37]. By imposing this constraint, we can ensure that the cell embedding has function mechanisms with biological significance.

**Task-specific joint optimization.** The task-specific objective for the annotation task serves as a weighted integration of the cross entropy loss and the auxiliary functional regularization: 


(11) \begin{align*}& \min_{\theta_{\mathrm{task}}} \mathcal{L}_{\mathrm{cls}} + \lambda_{\mathrm{path}} \mathcal{L}_{\mathrm{path}},\end{align*}

where $\theta _{\mathrm{task}}$ denotes all learnable parameters for cell type annotation, which includes the pretrained backbone and the task-specific functional heads. $\lambda _{\mathrm{path}}$ is the weighting factor that balances the classification and the auxiliary pathway prediction. This auxiliary task serves as a mechanism-aware regulator, incorporating biological inductive bias to enhance the generalizability and interpretability of cell identity predictions.

## Results

### Experimental settings and benchmarks

We pretrained scGenoByte on the large-scale datasets we integrated and then evaluate our method by comparing it with nine state-of-the-art approaches. To ensure the fairness and robustness of our results, we adopt a K-fold (K = 5) cross-validation strategy to evaluate. For down-stream cell annotation, we selected eight benchmark datasets covering diverse tissues including pancreas, lung, liver, PBMC. Specifically, in each fold, the testing data are strictly isolated and not involved in the training process. Furthermore, these benchmark datasets are excluded from the pretraining corpus. The information of these datasets is summarized in Table 1. Further details about datasets and evaluation metrics are provided in Supplementary Materials S1 and S3.

**Table 1:** Summary of downstream datasets used for evaluation.

Dataset	# Cells	# Genes	Cell types	Technology
Zheng68k [38]	68 450	16 906	11	10X Chromium
Baron [39]	8569	20 125	14	inDrop
Lung [40]	39 778	32 738	9	10X Genomics
Segerstolpe [41]	2128	22 757	12	SMART-Seq2
MacParland [42]	8439	20 007	13	10X Chromium
Xin [43]	1600	39 851	4	SMARTer
Muraro [44]	2119	18 915	9	CEL-Seq2
Pan-GI [45]	155 000	18 485	31	10X Smart-seq2

### Implementation details

All baseline models are obtained from their official repositories, ensuring reproducibility and consistency. To guarantee consistent input data quality, we evaluate all competing methods using the same data preprocessing pipeline, including quality control, gene expression normalization, and logarithmic transformation. The computational framework is implemented on the PyTorch version is 2.5.1 and completed on RTX 4090 GPUs. The regularization hyperparameters are set to $\lambda _{topo}=0.1$ and $\lambda _{path}=2.0$. For reproducibility of our method, the specific model parameters, hyperparameters, and evaluation metrics have been detailed in the attachment and code.

### Performance evaluation and analysis

#### Biological priors guided GenoByte

**Figure 2:**
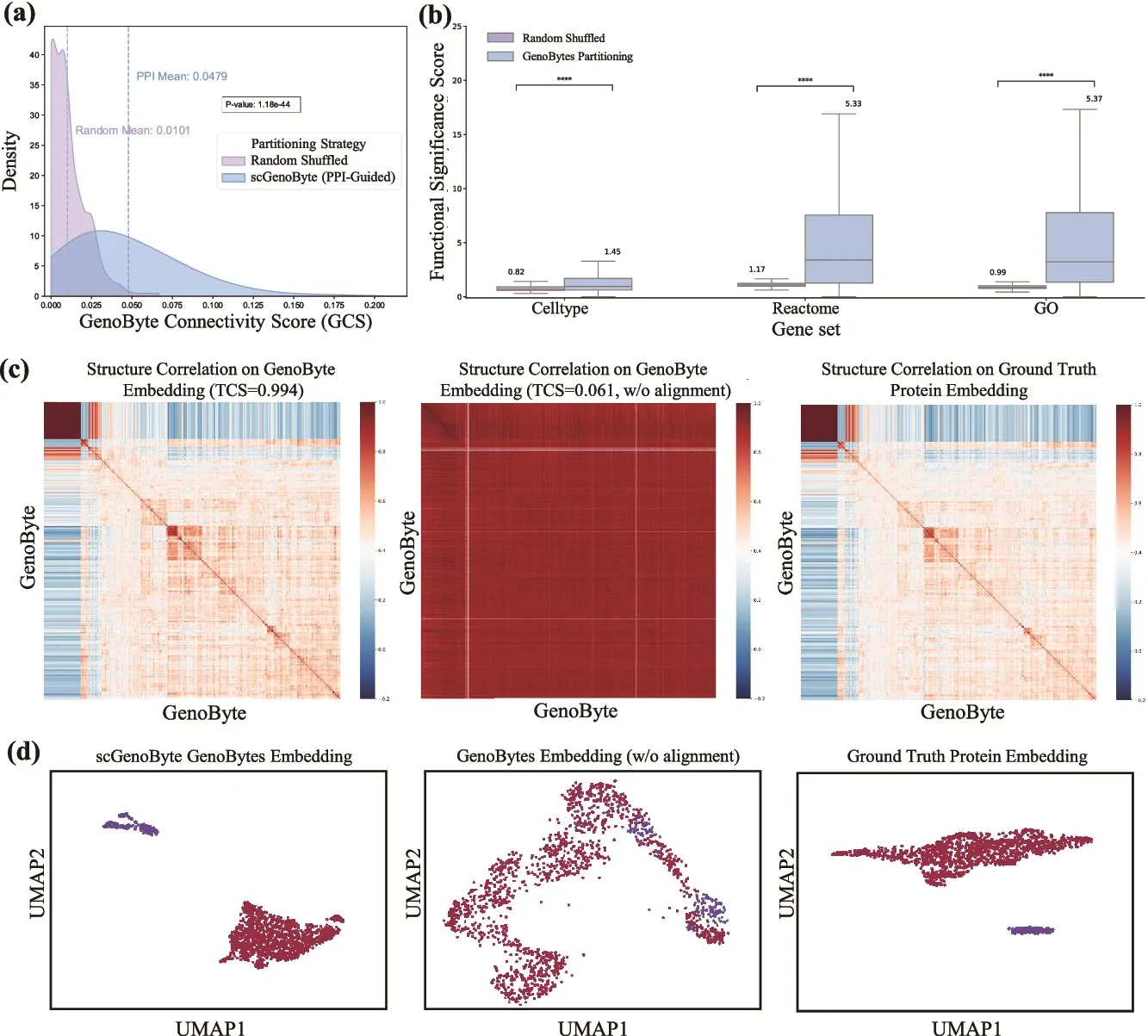
Validation of the biological priors guided GenoByte strategy. (a) Topological cohesiveness analysis: the density plots of gene connectivity scores are calculated based on the STRING experimental PPI scores with threshold *τ =100*. (b) Functional coherence verification: GenoBytes are significantly more enriched in biological genesets. (c) Topological alignment quantification: heatmaps display the cosine similarity matrices of embeddings. (d) UMAP visualization of the GenoByte embedding, where each dot represents a GenoByte.

To assess the biological cohesiveness of GenoBytes independent of the computational priors used in model construction, we re-evaluated the GenoByte Connectivity Score using a strictly experimental protein interaction network. We retrieved interactions from the STRING database (v12.0) [46], filtering exclusively for the “Experimental” evidence scores with a confidence threshold *τ =100*, which excludes all text-mining or prediction-based associations. As illustrated in Fig. 2a, scGenoByte exhibits a significantly higher density of interactions (Mean: *0.0479*) compared with random shuffles (Mean: *0.0101*), with a *P*-value of *1.18e-44*. The threshold *τ * denotes the filtering cutoff for the experimental evidence channel in the STRING database, ensuring that the connectivity analysis is strictly grounded in physical interactions with verified experimental support. This noncircular validation confirms that GenoBytes encapsulate biological priors rather than algorithmic artifacts.

Beyond embedding structural connectivity, we employed the functional significance score (*FSS*) to evaluate the enrichment of gene sets retrieved from the Molecular Signatures Database [47, 48]. Our evaluation specifically encompasses Reactome collection [35], the Gene Ontology collection [49], and the cell type signature collection. As shown in Fig. 2b, GenoBytes achieves higher enrichment scores, particularly in the Reactome ( *FSS=5.33*) and GO ( *FSS=5.37*) genesets. This indicates that genes within a single GenoByte are highly likely to participate in the specific biological process or signaling pathway.

#### Validation of protein-informed semantic alignment

To verify the effectiveness of our semantic alignment mechanism, we conducted a comparative analysis of the embeddings learned by scGenoByte and the protein representations derived from pretrained protein language model ESM-2.

We first employed representational similarity analysis to quantitatively evaluate the topological isomorphism between the two modalities. We visualized the cosine similarity matrices of the learned GenoByte embeddings and the ground-truth protein embeddings in Fig. 2c. Without semantic alignment, the similarity matrix appears chaotic, resulting in a low topological consistency score (*TCS*) score as *0.061*. After integrating $\mathcal{L}_{topo}$, the embeddings similarity structure of scGenoByte is highly consistent with the protein embeddings from ESM-2, achieving the TCS value of 0.994. This high degree of correlation indicates that scGenoByte has retained the inherent pairwise relationships and higher-order representation information in the protein space.

Furthermore, to intuitively assess the alignment between transcriptomic and proteomic representations, we visualized the embedding distributions [50]. As shown in Fig. 2d, the embeddings generated by scGenoByte (left panel) exhibit distinct structural features, indicating its ability to recover the topological information within protein sequences. In contrast, in the absence of alignment (middle panel), the transcriptomic embeddings appear as a diffuse and unstructured cloud, failing to capture functional boundaries. These results demonstrate that scGenoByte successfully aligns transcriptomic representations with the well-defined structure of protein embeddings from ESM-2 (right panel).

#### Cell annotation with pathway-guided regularization

**Figure 3:**
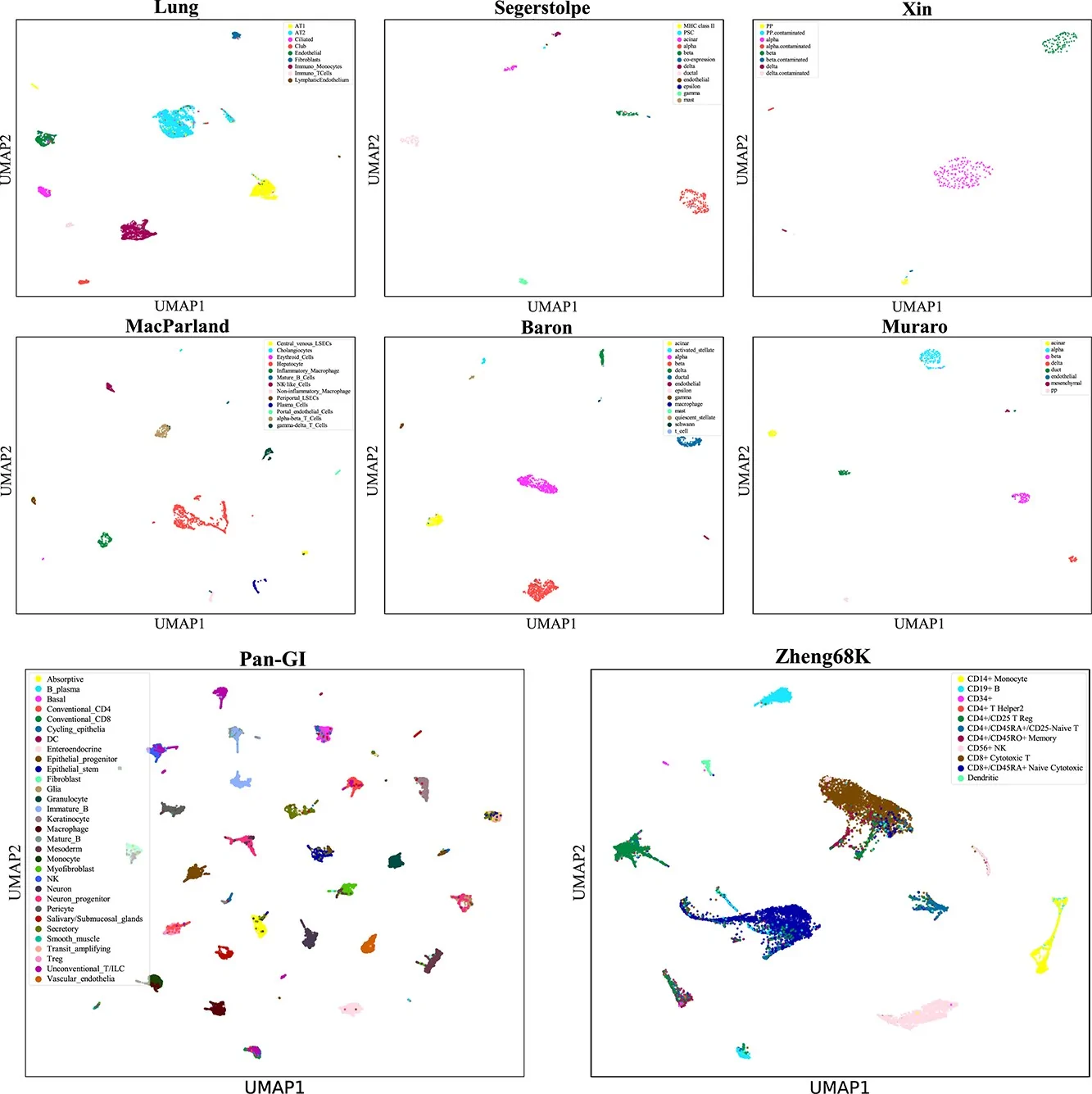
Visualization of cellular representations learned by scGenoByte. Each dot represents an individual cell. The UMAP visualization is constructed using the high-dimensional cell embeddings ($h_{cell}$) learned by the scGenoByte encoder.

We evaluated the model’s performance on cell type annotation against nine competing methods. The quantitative results are summarized in Table 2. scGenoByte consistently outperforms all baselines across datasets of varying scale and complexity. All eight benchmark datasets are clearly verified established through rigorous experimental protocols such as FACS, IHC, or smFISH to ensure reliable ground-truth labels. Notably, on the FACS-verified gold standard Zheng68k dataset, scGenoByte achieved an ACC of 0.863. Moreover, on the Pan-GI dataset, which presents a significant challenge due to its high heterogeneity [51, 52], scGenoByte achieves an accuracy of *0.923* and an F1-score of *0.921*, surpassing the second-best model scGPT by over *2%*. This demonstrates that integrating GenoByte-level structural information and protein-level semantic constraints significantly boosts the model’s ability to distinguish fine-grained cell subtypes. To qualitatively assess the quality of the learned embeddings, we visualized the latent space using UMAP on the test datasets (Fig. 3). The plots reveal that scGenoByte generates highly cohesive and well-separated clusters. Even for closely related subtypes in Pan-GI and Zheng68k, scGenoByte retains their local neighborhood structure. The results demonstrate the effectiveness of the GenoByte construction strategy and its contribution to robust and discriminative cell representation learning.

**Table 2:** Quantitative comparison of cell type annotation performance.

**Methods**	**Pan-GI**		**Zheng68k**		**Muraro**		**Xin**		**Segerstolpe**		**MacParland**		**Lung**		**Baron**	
	ACC *↑ *	F1 *↑ *	ACC *↑ *	F1 *↑ *	ACC *↑ *	F1 *↑ *	ACC *↑ *	F1 *↑ *	ACC *↑ *	F1 *↑ *	ACC *↑ *	F1 *↑ *	ACC *↑ *	F1 *↑ *	ACC *↑ *	F1 *↑ *
scNym	0.892	0.891	0.697	0.621	0.952	0.799	0.899	0.542	0.833	0.648	0.962	0.959	0.925	0.872	0.960	0.801
SciBet	0.822	0.812	0.679	0.667	0.947	0.812	0.979	0.791	0.793	0.728	0.971	0.957	0.909	0.847	0.971	0.867
Seurat	0.789	0.788	0.686	0.581	0.954	0.851	0.959	0.594	0.846	0.652	0.979	0.965	0.901	0.833	0.961	0.833
scmap	0.761	0.734	0.463	0.482	0.892	0.752	0.956	0.780	0.602	0.619	0.931	0.899	0.893	0.717	0.912	0.826
scBERT	0.818	0.790	0.759	0.691	0.956	0.948	0.980	0.793	0.892	0.759	0.976	0.959	0.957	0.914	0.962	0.849
SCTrans	0.834	0.834	0.814	0.712	0.961	0.959	0.995	0.946	0.977	0.826	0.979	0.964	0.957	0.917	0.981	0.883
scGPT	0.898	0.897	0.846	0.752	0.970	0.971	0.992	0.980	0.982	0.911	0.981	0.970	0.964	0.940	0.975	0.961
scGraphformer	0.834	0.820	0.713	0.552	0.974	0.970	0.978	0.703	0.991	0.980	0.970	0.962	0.946	0.885	0.978	0.931
scFoundation	0.854	0.851	0.830	0.733	0.968	0.965	0.988	0.922	0.972	0.959	0.971	0.961	0.921	0.862	0.974	0.937
**scGenoByte**	**0.923**	**0.921**	**0.863**	**0.770**	**0.989**	**0.986**	**0.999**	**0.999**	**0.998**	**0.994**	**0.986**	**0.982**	**0.974**	**0.947**	**0.989**	**0.991**

**Figure 4:**
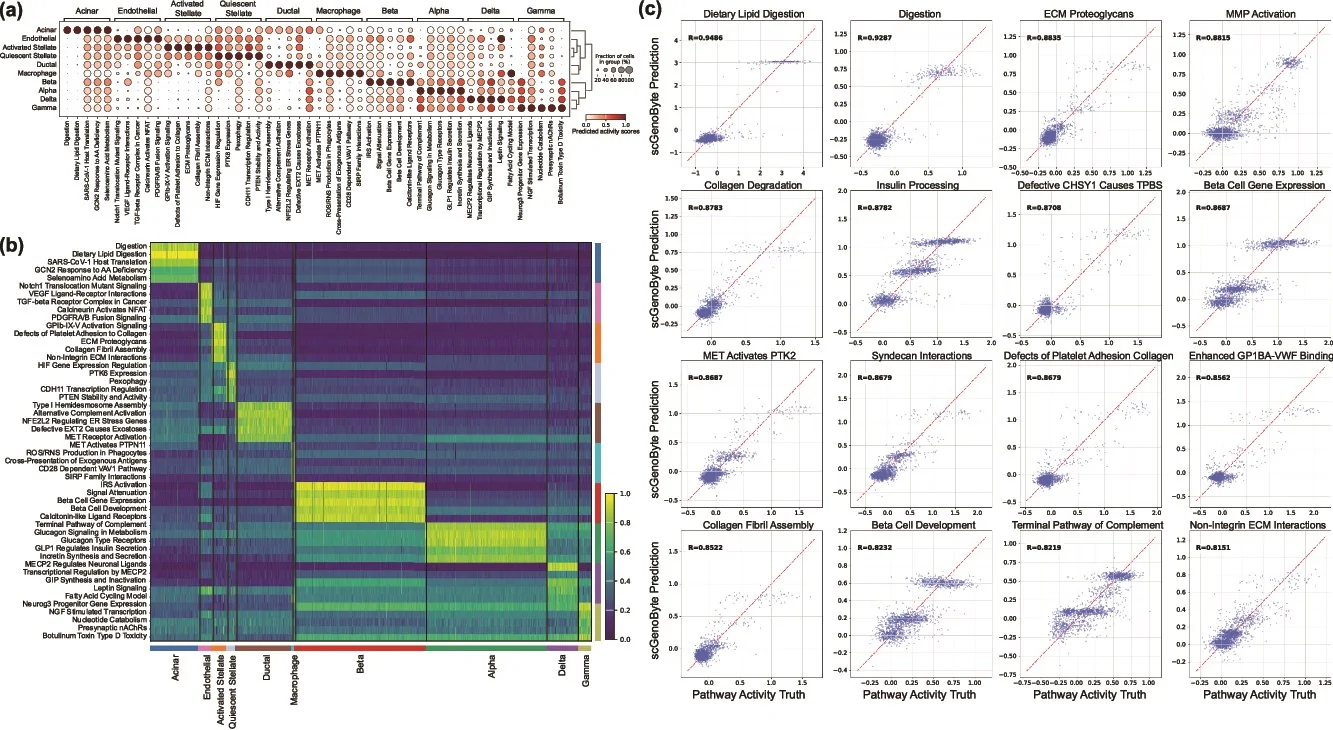
Performance of the auxiliary pathway activity prediction task on the Baron dataset. (a) Bubble plot showing the enrichment of the top five cell-type-specific pathways. (b) Heatmap visualization of the predicted activity scores for cell-type-specific pathways, showing clear block-diagonal patterns consistent with cell identity. (c) Scatter plots comparing the predicted pathway scores with ground-truth activity scores derived from ssGSEA for 16 marker pathways, selected by taking the top specific pathways for each cell type in the Baron dataset. The Pearson correlation coefficient (*R*) is shown for each plot, indicating high predictive accuracy.

A unique feature of scGenoByte is its ability to infer biological pathway activities simultaneously with cell classification. We validated this capability on the Baron pancreas dataset, as shown in Fig. 4. For each cell type, we selected the top five marker pathways and visualized the activity prediction scores. The bubble plot (Fig. 4a) and heatmap (Fig. 4b) demonstrate that scGenoByte correctly identifies cell-type-specific functional signatures. For instance, in Acinar cells, the model predicts high activity for “Digestion” and “Dietary Lipid Digestion” pathways, which aligns perfectly with their physiological role as exocrine cells responsible for secreting digestive enzymes [53]. Similarly, for Beta cells, scGenoByte identifies “Beta Cell Gene Expression” and “Beta Cell Development” as top functional markers, consistent with their specialized endocrine function in insulin production and glucose homeostasis [54, 55]. Furthermore, we evaluated the prediction accuracy for 16 representative pathways in Fig. 4c. The scatter plots show a strong linear correlation between the predicted scores and the ground-truth activity scores derived from ssGSEA, with Pearson correlation coefficients (R) consistently exceeding *0.85*. This high correlation confirms that scGenoByte learns a mechanistic understanding of cellular functions.

#### Analysis of annotation and functional alignment

To provide a granular understanding of the classification boundaries and the biological validity of the learned representations, we visualized the misclassification networks and the functional correlation heatmap on the Pan-GI dataset. This analysis aims to dissect the nature of the errors and verify whether the model captures the underlying functional logic of cellular identities.

**Figure 5:**
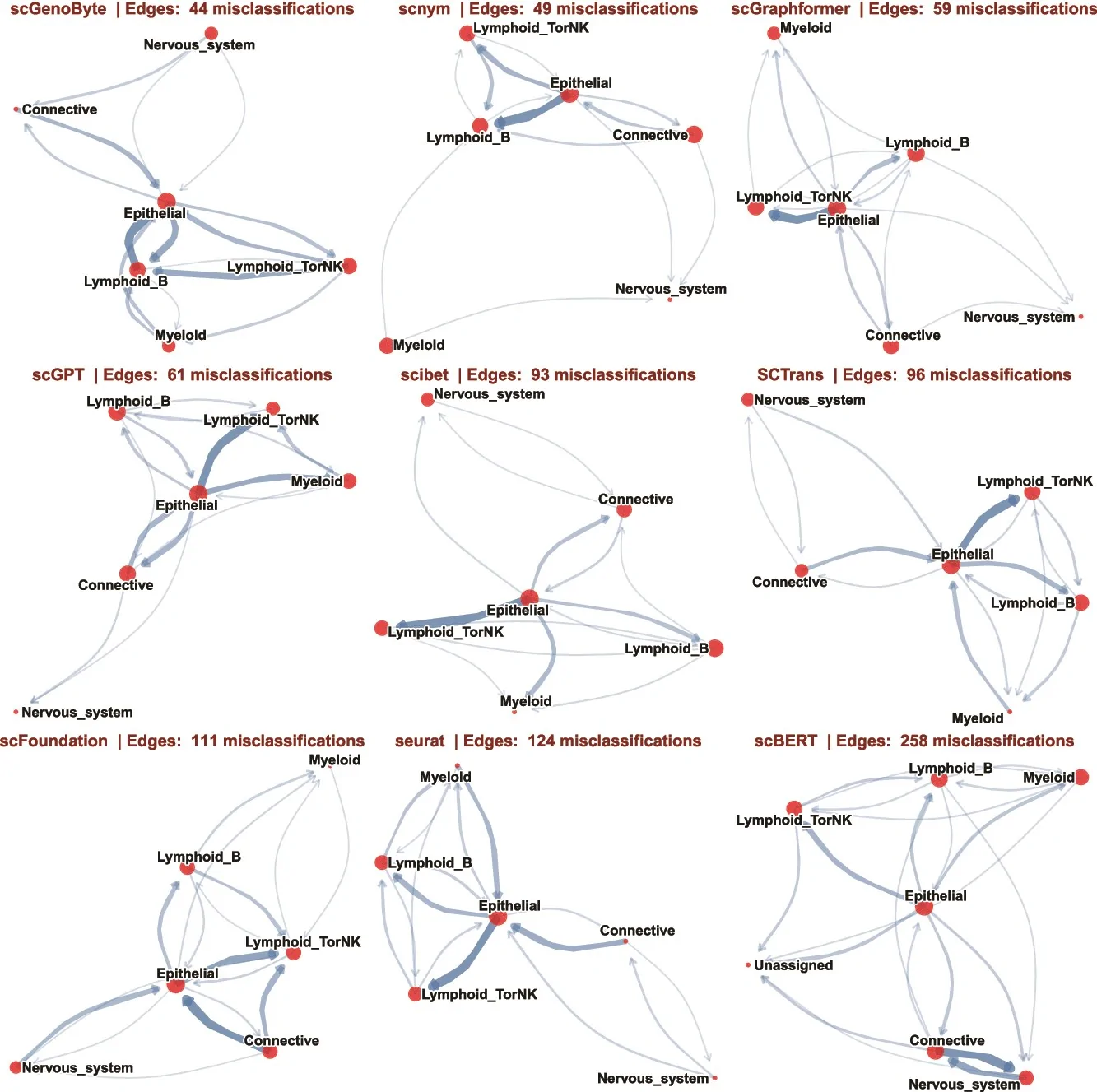
Maintype misclassification networks illustrating coarse-grained confusion patterns.

**Figure 6:**
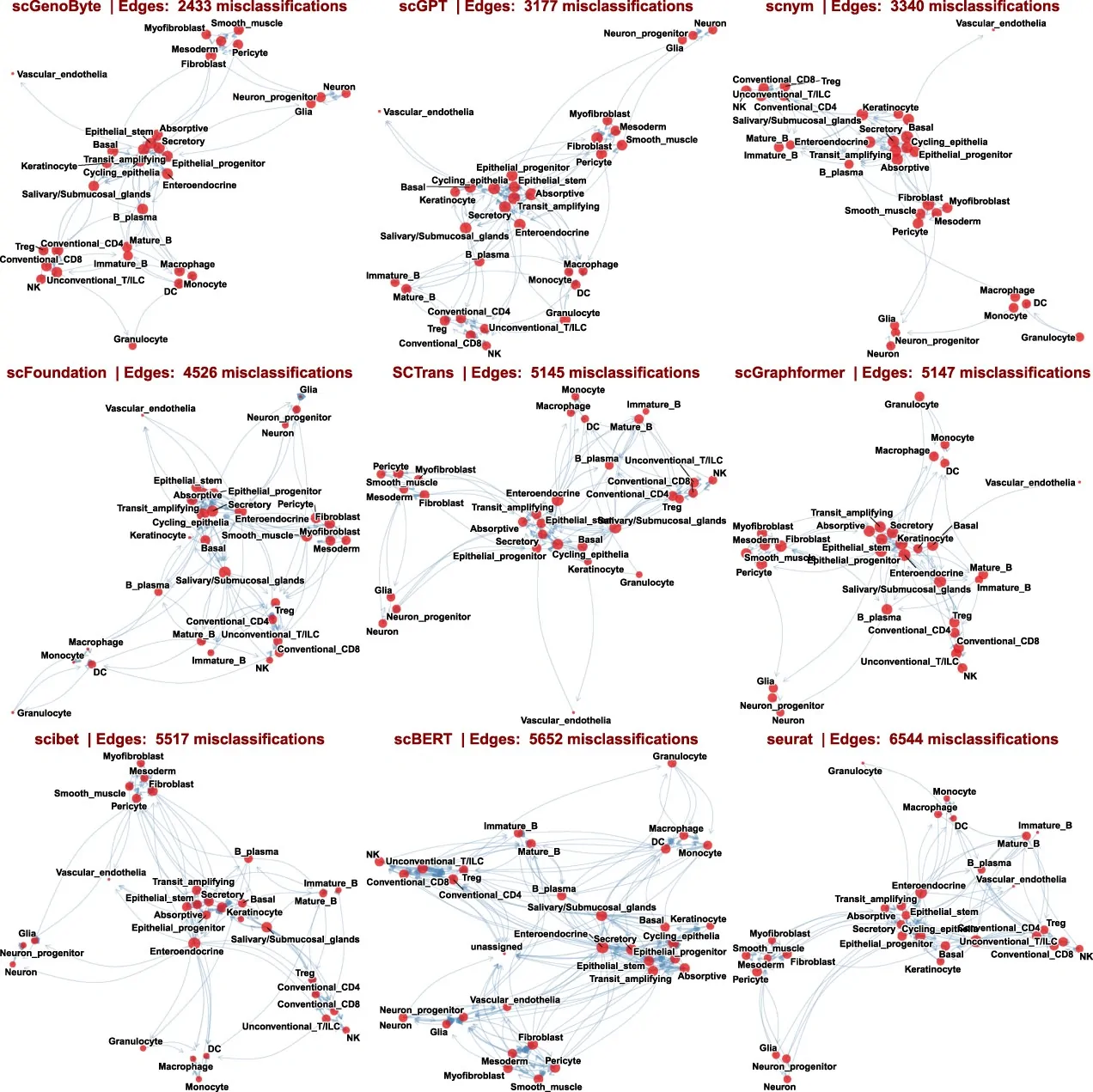
Subtype misclassification networks revealing lineage-constrained errors.

We first examined the robustness of classification boundaries through network topology analysis. To visualize the specific patterns of error, we constructed misclassification networks where nodes represent ground-truth cell types. In these graphs, a directed edge signifies a misclassification event, originating from the true cell identity and pointing to the incorrectly predicted category, with the edge width proportional to the number of error samples. As illustrated in Fig. 5, scGenoByte demonstrates a marked reduction in confusion frequency, narrowing the total misclassification count to 44. Moreover, this robustness extends to the subtype level where cells are intrinsically more difficult to distinguish. At the level of subtype annotation, scGenoByte minimizes the error count to 2433, as shown in Fig. 6, significantly lower than the second-best method (3177 errors). In addition, these errors are mainly concentrated within biological clusters, such as the epithelial progenitor-stem cell continuum, indicating that scGenoByte retains the hierarchical information of cell identity even when errors occur.

**Figure 7:**
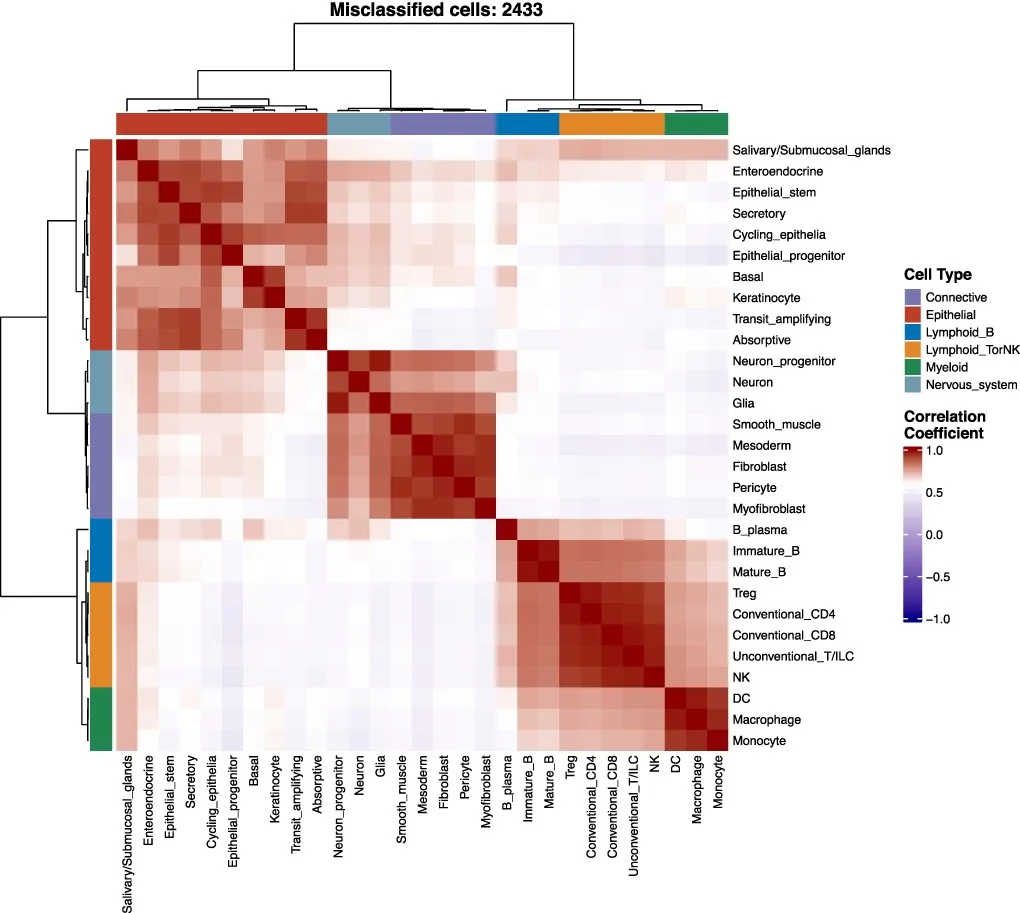
Evaluation of functional consistency via pathway activity correlation on misclassification samples.

Furthermore, to validate the functional coherence of the predictions, we analyzed the correlation of model-inferred pathway activity scores across cell types as shown in Fig. 7). By computing the pairwise Spearman correlation of these functional profiles, we observed distinct, high-intensity block-diagonal patterns corresponding to major cell lineages. Within these blocks, biologically related subtypes exhibit strong functional correlations, distinct from unrelated populations. This indicates that the inferred pathway enrichment patterns faithfully reflect the intrinsic functional attributes of the cells.

#### Ablation study

To rigorously verify the contribution of each proposed component in scGenoByte, we conducted ablation studies focusing on three key modules, as shown in Table 3. The baseline configuration is set to randomly partition the gene sub-vectors, achieving an accuracy of 0.882. After adopting GenoBytes, the accuracy of scGenoByte increased by 2.0% to 0.902, demonstrating that PPI networks and paralogy information are essential for functional semantics. Building on this foundation, the subsequent integration of semantic alignment further bridges the transcriptomic-proteomic pattern gap, and lifts accuracy to 0.909. In addition, the full scGenoByte framework achieves optimal performance by incorporating auxiliary pathway regularization, which indicates that mechanism-aware regularization serves as a potent constraint.

**Table 3:** Ablation study of key components on the Pan-GI dataset.

GenoByte partitioning	Protein-informed semantic alignment	Auxiliary pathway prediction task	ACC *↑ *	F1 *↑ *
-	-	-	0.882	0.876
✓	-	-	0.902	0.895
✓	✓	-	0.909	0.901
✓	✓	✓	**0.923**	**0.921**

## Conclusion

In conclusion, we present scGenoByte, a novel single-cell transcriptome foundation model that harmonizes complete transcriptome context with biological priors. Our framework addresses the long-standing trade-off between genomic coverage and computational scalability. Extensive results demonstrate that scGenoByte not only achieves state-of-the-art performance, but also encodes embedding vectors with substantial biological semantic information.

Looking forward, scGenoByte is poised to serve as a robust baseline for the single-cell community. Effective whole-genome modeling is meaningful for single-cell transcriptome analysis. As for cell representation learning, it necessitates not only data-driven modeling but also meaningful alignment with biological priors. It is convinced that further research efforts could extend our framework to diverse down-stream tasks and adapt to more effective biological priors. Specifically, future developments may incorporate direct phenotypic biomarkers or products of interlinked signaling dynamics to capture the cellular functional landscape with even greater granularity. Furthermore, leveraging reinforcement learning for dynamic state abstraction represents a promising direction, enabling the model to adaptively optimize partitioning resolution.

**Key points**

- We proposed scGenoByte, a novel transformer-based single-cell foundation model that integrates genome-wide modeling and biological priors.
- We designed GenoBytes, novel tokenization units constructed by leveraging gene homology information and protein interaction networks.
- The protein-informed semantic alignment integrates protein evolutionary semantic information into GenoByte, providing critical protein context for representation.
- An auxiliary pathway activity prediction task is introduced to regularize the model, enhancing representation learning and biological interpretability.
- Extensive results on diverse datasets demonstrates superior performance of scGenoByte over competing methods, highlighting accurate cell annotation and robust generalization capabilities.

## Supplementary Material

Supplementary_Material_bbag369

## Data Availability

The code and the datasets underlying this paper are available at our github repository https://github.com/yjs193/scGenoByte.
