# Supplementary material for: scGenoByte: a GenoByte embedding transformer with biological priors for cell type annotation
Source: Brief Bioinform. 2026 Jul 6;27(4):bbag369. doi: 10.1093/bib/bbag369 (PMC13336651; doi:10.1093/bib/bbag369)
Supplement: Supplementary_Material_bbag369 [file supplementary_material_bbag369.pdf]

Supplementary Materials

A GenoByte Embedding Transformer with Biological Priors for Cell Type Annotation

Jiongsen Yao[1], Yong Xu[1], Jinjin Ma[3], Wenjun Shen[*2], and Si Wu[*1]

[1]School of Computer Science and Engineering, South China University of Technology, Guangzhou, 510006, China

[2]Department of Bioinformatics, Shantou University Medical College, Shantou, 515041, China

[3]The Institute of Future Health, South China University of Technology, Guangzhou, 511442, China

S1 Dataset Details

Pre-training dataset. To enable scGenoByte to learn robust and biologically meaningful cellular representations, we constructed a comprehensive large-scale pre-training corpus. We initially aggregated approximately 3 million single-cell transcriptomic profiles by integrating 303 distinct datasets spanning more than 50 human tissues, sourced from *PanglaoDB*[1]. After applying rigorous data cleaning and quality control procedures to remove low-quality cells and potential artifacts, a refined pretrain dataset of 1.2 million cells was retained for the pre-training phase. PanglaoDB is a widely recognized database that collects and standardizes single-cell RNA sequencing data from mouse and human tissues, providing a rich resource for investigating cellular heterogeneity and gene expression dynamics. By leveraging this massive, cross-tissue dataset, scGenoByte captures the underlying gene expression patterns and gene-gene co-occurrence relationships common across different biological contexts.

Downstream datasets. To rigorously evaluate the performance of scGenoByte, we selected eight benchmark datasets covering diverse tissues (e.g., Pancreas, Lung, Liver, PBMC) and sequencing technologies (e.g., 10X Chromium, SMART-Seq2, inDrop).

Distinguishing subtle differences between cell subtypes poses a greater challenge. Therefore, we specifically utilize the Zheng68k and Pan-GI datasets to assess the model's capability in cell subtype annotation. Notably, the Pan-GI dataset is a large-scale collection derived from the gastrointestinal tract, featuring high cellular diversity. To construct a balanced dataset for rigorous evaluation, we sampled 5,000 cells for each of the 31 distinct cell subtypes, resulting in a total of 155,000 cells. This stratified sampling strategy ensures that the evaluation is not biased towards majority cell populations.

All of the preprocessed datasets for cell type annotation can be accessed from the repository or accessed from the link. Besides, the single-cell RNA-seq raw datasets analyzed in this work can be accessed via the following links: Zheng68k, the pancreatic suite (Baron, Muraro, Segerstolpe, and Xin), MacParland, Lung and Pan-GI.

*Corresponding authors: wjshen@stu.edu.cn; cswusi@scut.edu.cn

[1]`https://panglaodb.se/`

S2 Algorithm for Resolution-adaptive Iterative Clustering

To partition the gene set $\mathcal{V}$ into biologically coherent GenoBytes, we employ a resolution-adaptive iterative clustering strategy based on the Leiden framework. While standard modularity optimization often produces clusters with significant size heterogeneity, our approach ensures each GenoByte $\mathcal{B}_k$ satisfies strict cardinality constraints: $|\mathcal{B}_k| \in [Size_{\min}, Size_{\max}]$, where $Size_{\min} = 1$ and $Size_{\max} = 16$.The algorithm initiates with a baseline resolution $\gamma = 1.0$. Any cluster exceeding $Size_{\max}$ is recursively decomposed using an adaptive resolution increment $\Delta\gamma = 0.5$. This hierarchical refinement ensures that GenoBytes encapsulate dense local topologies while maintaining the computational tractability required by the Transformer encoder.

Algorithm S1 Resolution-adaptive iterative clustering

Require: Gene set $\mathcal{V}$, Interaction matrices S_{PPI}, S_{Paralogy}
Require: Params: $\mathrm{Size}_{\min} = 1$, $\mathrm{Size}_{\max} = 16$, $\Delta\gamma = 0.5$, $\alpha = 0.2$
Ensure: Set of GenoBytes $\mathbb{B} = \{\mathcal{B}_1, \mathcal{B}_2, \dots\}$

Phase 1: Interaction graph construction
$w_{ij} \leftarrow S_{\mathrm{PPI}}(i,j) + \alpha \cdot S_{\mathrm{Paralogy}}(i,j)$ ▷ Calc. affinity weights
$\mathcal{G} \leftarrow (\mathcal{V}, \mathcal{E}, \mathcal{W})$ ▷ Init. graph topology

Phase 2: Hierarchical refinement
$\mathcal{Q} \leftarrow [(\mathcal{G}, 1.0)]$ ▷ Initialize processing queue
$\mathbb{B} \leftarrow \emptyset$
while $\mathcal{Q} \neq \emptyset$ **do**
  $(\mathcal{G}_{curr}, \gamma) \leftarrow$ Dequeue($\mathcal{Q}$)
  $\{C_1, \dots, C_k\} \leftarrow$ Leiden($\mathcal{G}_{curr}, \gamma$) ▷ Apply Leiden partitioning
  if $k = 1 \wedge |C_1| > Size_{\max}$ **then**
    Enqueue($\mathcal{Q}$, $(\mathcal{G}[C_1], \gamma + \Delta\gamma)$) ▷ Refine with higher resolution
  else
    for each cluster $C_j \in \{C_1, \dots, C_k\}$ **do**
      if $|C_j| > Size_{\max}$ **then**
        Enqueue($\mathcal{Q}$, $(\mathcal{G}[C_j], 1.0)$) ▷ Recurse on sub-graph
      else if $|C_j| \geq Size_{\min}$ **then**
        $\mathbb{B} \leftarrow \mathbb{B} \cup \{C_j\}$ ▷ Save valid GenoByte
      end if
    end for
  end if
end while
return $\mathbb{B}$

S3 Formal Definitions of Metrics

To comprehensively assess the performance of scGenoByte across cell type annotation, we utilize accuracy (ACC) and Macro F1-score ($F1$). ACC quantifies the overall proportion of correctly classified cells. Let N be the total number of cells, y_i be the true label of the i-th cell, and $\hat{y}_i$ be the predicted label. ACC is defined as:

$$ACC = \frac{1}{N} \sum_{i=1}^{N} \mathbb{I}(\hat{y}_i = y_i), \tag{S1}$$

where $\mathbb{I}(\cdot)$ is the indicator function. However, single-cell datasets often exhibit significant class imbalance. To address this, we employ the Macro F1-score, which treats all cell types equally regardless of their size. It is calculated by averaging the F1-scores of each class c:

$$F1 = \frac{1}{C}\sum_{c=1}^{C}\frac{2 \cdot P_c \cdot R_c}{P_c + R_c} \tag{S2}$$

where C is the number of cell types, and P_c and R_c represent the precision and recall for the class c, respectively. This ensures a balanced assessment of the model's ability to identify rare cell populations.

To evaluate the biological interpretability of the proposed GenoByte strategy, we introduce three specialized metrics. First, the functional significance score (FSS) quantifies the statistical significance of biological pathway enrichment within a GenoByte. It is derived from the gene set enrichment analysis results and is defined as follows:

$$FSS = -\log_{10}(\min(\text{Adj. } P_{val}) + \epsilon), \tag{S3}$$

where $\min(\text{Adj. } P_{val})$ denotes the minimum adjusted p-value across tested pathways, and ϵ is a small constant for numerical stability.

GenoByte Connectivity Score (GCS) measures the internal protein-protein interaction density. For a GenoByte B of size N, GCS represents the ratio of observed edges to the maximum possible connections, defined as:

$$GCS = \frac{2 \cdot \sum_{i,j \in B, i<j} \mathbb{I}(\text{score}_{ij} > \tau)}{N(N-1)}, \tag{S4}$$

where $\mathbb{I}(\cdot)$ is an indicator function that equals 1 only if the PPI confidence score between genes g_i and g_j exceeds a predefined threshold τ, thereby establishing a valid edge in the network.

Finally, the Topological Consistency Score (TCS) assesses the alignment between the learned GenoByte embeddings and the ground-truth protein representations using representational similarity analysis. It is calculated as the Pearson correlation between their similarity structures:

$$TCS = \rho(\text{vec}(S_{pred}), \text{vec}(S_{truth})) \tag{S5}$$

where $\rho(\cdot, \cdot)$ denotes the Pearson correlation coefficient. S_{pred} and S_{truth} represent the cosine similarity matrices of the predicted GenoByte embeddings and the ground-truth protein embeddings, respectively, and $\text{vec}(\cdot)$ signifies the vectorization of the upper triangular elements.

To assess the model's efficiency, we measure the computational cost using Floating Point Operations (FLOPs). FLOPs provide a hardware-independent quantification of the model's theoretical complexity, which is crucial when varying the GenoByte size. In our analysis, we report the normalized FLOPs to demonstrate the trade-off between the granularity of GenoBytes and the computational overhead of the transformer backbone.

S4 Parameter Analysis

S4.1 Impact of masking ratio

The masking ratio r represents a pivotal trade-off between the difficulty of self-supervised reconstruction and the integrity of the contextual signal. As illustrated in Figure S1, scGenoByte exhibits a robust operational window within the range of $[0.500, 0.750]$, where both ACC and $F1$-score maintain a stable and high-performance plateau. Within this optimal regime, the model demonstrates remarkable resilience to varying degrees of data occlusion, suggesting that it effectively captures the underlying gene regulatory logic rather than over-relying on local sparsity patterns.

For all subsequent analyses, we adopted 0.625 as the default configuration. This value serves as a representative setting within the high-performance plateau, providing an ideal equilibrium that sufficiently challenges the model's inferential capacity while preserving the essential biological semantics required for robust representation learning.

S4.2 Impact of GenoByte size

The GenoByte size determines the granularity of gene grouping, representing a trade-off between biological resolution and computational efficiency. Smaller GenoBytes capture finer local gene interaction patterns but result in longer sequence lengths, thereby increasing the computational burden of the transformer's self-attention mechanism. Conversely, larger GenoBytes reduce computational cost but may lead to over-smoothing of gene features.

We investigated this trade-off by varying the GenoByte size from 8 to 100. As shown in Figure S2, while a size of 8 yields high accuracy, it incurs the highest computational cost. Increasing the size to 16 results in a negligible drop in accuracy but a sharp reduction in computational overhead. Further increasing the size leads to a substantial decline in model performance. Therefore, we selected a GenoByte size of 16 to achieve an optimal balance between accuracy and efficiency.

S5 Supplementary Figures

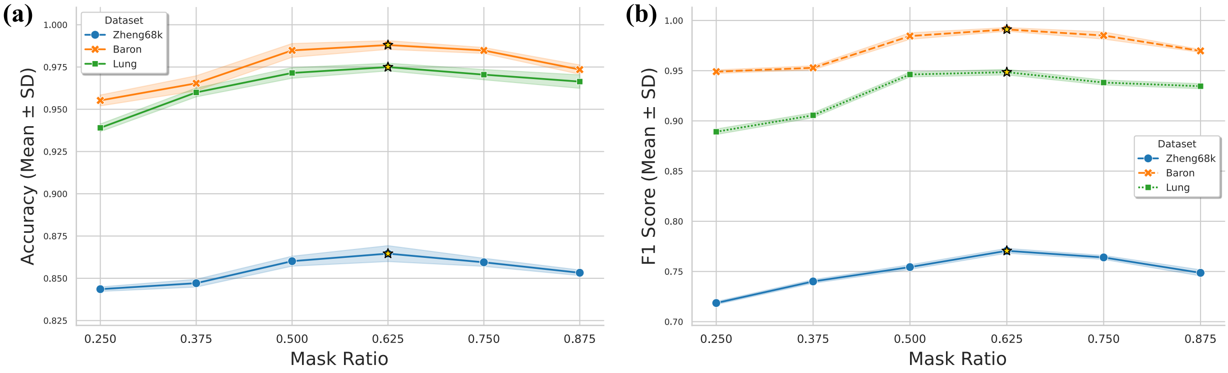

Figure S1: Impact of different masking ratios on model performance across Zheng68k, Baron, and Lung datasets. The star (⋆) denotes the optimal performance at ratio 0.625.

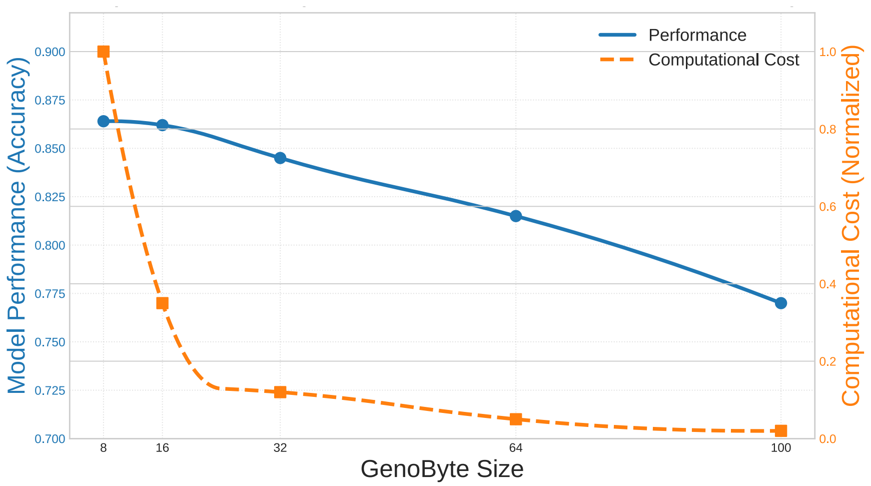

Figure S2: The trade-off between model performance and computational cost across different GenoByte sizes. A size of 16 offers the optimal balance.
